# A spectroscopic and quantum chemical calculation method for the characterisation of metal ions complexed with propyl gallate and procyanidins

**DOI:** 10.1038/s41598-023-30186-x

**Published:** 2023-02-20

**Authors:** Liangliang Zhang, Qinhao Guan, He Zhang, Lihua Tang, Man Xu

**Affiliations:** 1 Academy of Advanced Carbon Conversion Technology, Huaqiao University, Xiamen, 361021 China; 2 Institute of Chemical Industry of Forest Products, CAF, Nanjing, 210042 China; 3 Institute of Biomedical Health Technology and Engineering, Shenzhen Bay Laboratory, Shenzhen, 518107 China

**Keywords:** Fluorescence spectroscopy, Spectrophotometry, Quantum chemistry

## Abstract

The deprotonation mechanism for the phenolic hydroxyl and the complexing of metal ions with a commonly used food additive, propyl gallate (PG) were studied theoretically and experimentally. The interaction of procyanidins [PC, epicatechin_16_ (4 → 8) catechin], and its basic monomeric unit catechin (CA) with metal ions was studied by the fluorescence quenching spectra. The results showed that the 9-OH quinoid PG was formed at higher pH (10.9) by the oxidization of phenolic hydroxyl. The binding affinities (*K*_a_) and stoichiometry of these metal ions with PG were determined. The Al^3+^ in PG-Al complex [Al(PG)(H_2_O)_2_Cl_2_]^-^ was coordinated at the 8,9-OH doubly deprotonated catechol site with double chloride ions (Cl^-^) and double water molecules (H_2_O). The fluorescence quenching titration with Sn^2+^, Zn^2+^, Cu^2+^, Al^3+^ and Fe^3+^ revealed that the stoichiometries of metal-bound PC were 1:1, 2:3, 2:3, 2:3 and 1:1, respectively. The presence of bovine serum albumin (BSA) could enhance the complexing strength of PC with metal ions.

## Introduction

**Figure 1:**
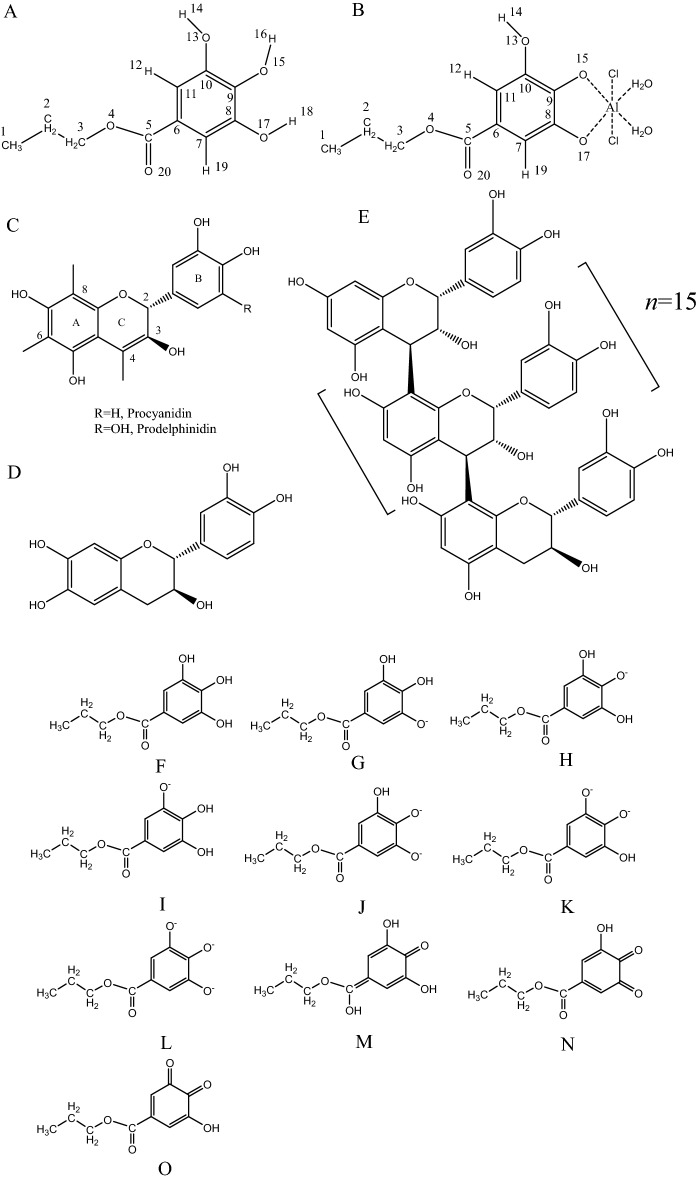
Molecular structures of (**A**) propyl gallate (PG), (**B**) PG-Al complex, (**C**) flavan-3-ol monomer of condensed tannin, (**D**) catechin and (**E**) PC; Propyl gallate (PG), proposed deprotonated and quinoid PG, and PG-Al complex structures used to calculate spectra by Gaussian software(**F**–**O**).

Propyl gallate (PG, Fig. 1A) is the *n*-propyl ester of gallic trihydroxybenzoic acid propyl ester^1^. As an antioxidant, PG was generally utilized to prevent rancidity and spoilage in processed cosmetics, food and related packing materials. It was on the US Food and Drug Administration list for preservation and stabilization of pharmaceutic preparations^2^. Furthermore, PG may contain some physiological activities on the functions of tissue and cell. Many advantages of PG were studied. For example, as an antioxidant and as a superoxide dismutase mimic, PG protected cultured lens epithelial cells from H_2_O_2_ insult^3^. PG contained topical anti-inflammatory activity in a mouse ear edema test^4^. Recently, PG induced apoptosis in a large variety of cancer cells^5^. In addition, PG is usually added to food as an antioxidant due to its excellent antioxidant capacity, and its antioxidant mechanism has also been widely studied^6,7^. The anticancer activity and protein binding ability of propyl gallate metal complexes have also been a research hotspot^8^.

The antioxidant activities of polyphenols and their derivatives were closely related to the biding number and site, (mutual) position of phenolic hydroxyl groups, and the nature of substituents^9^. The *ortho*-diphenols possessed the highest antioxidant ability^10^. The *ortho*-dihydroxy polyphenols (for example, molecules with catechol or galloyl groups) were supposed to mainly contribute to the metal chelating capability of polyphenols^11^. The metal chelation is physiologically significant due to its occurrence in the physiological pH range. Chelation therapy is a preferable method to reduce the toxicity from heavy metals. The complex structures of tightly binding toxic metal ions to the chelating agents are easily excreted from the body. Some polyphenols were used to chelate transition metals so as to modulate physiological reactions^12^. Previous studies demonstrated that the chelation of metal ions with phenolic compounds preferred to occur at the deprotonated catechol sites^13–15^. The good antitumor activities of PG accounted for the complexation of metal cations such as platinum and ruthenium^16^. However, to the best of our knowledge, the complexation mechanism of metal ions with PG has not been fully elucidated yet. Moreover, the effect of tannin structure on the binding reaction with metal ions has not been studied thoroughly. Quantitative study was lacking on the interactions between metals and tannins, especially the pure and chemically defined tannins.

Tannins and lignins are the most abundant polyphenols produced in plants. There are usually two kinds of tannins, that is, hydrolysable tannins (HTs) and condensed tannins (CTs), often called proanthocyanidins. HTs usually consist of a sugar core (normally glucose) surrounded by gallic acid or ellagic acid moieties. CTs are polymers and oligomers of flavan-3-ol units, most often lined either via C_4_-C_6_ bonds (A-type proanthocyanidins) or C_4_-C_8_ (B-type proanthocyanidins) (Fig. 1C). Generally, CTs are composed of phloroglucinol-type A-rings, such as procyanidins and prodelphinidins. Due to the nonadjacent hydroxyl groups of phloroglucinol-type A-rings, the metal chelating ability of A-rings is less important than that of B-rings. The procyanidins (PC), the most common CTs in plant tissues, are derived from catechin or epicatechin, and PC often contains gallic acid esters^17^. Through the induction of some physiological effects, PC can interact with biological systems^17^.

Polyphenols can retard decomposition and mineralization processes of organic matter^18^ and can form strong interactions with soil mineral nutrients, organic matter and free metals (such as Fe and Al)^19^. Complexing with metal cations (such as Fe^3^^+^) is important for both microorganism growth and plant defense against biotic aggressors^20^. These properties are also of significance in the fields of human nutrition and health, because of their contribution to the decreased gastrointestinal absorption of certain essential metal cations^21^. Many applications of tannins or tannin-containing materials in industry are dependent on the chelating properties of metal ions. Complexing polyphenols with metal ions was widely employed to analyze both metals and polyphenols^22^ in the fields of agriculture, corrosion inhibition, leather tanning and water treatment. To the best of our knowledge, the polyphenol-metal complexes have been extensively researched for practical application, but the mechanism of formation of relevant polyphenol-metal complexes has been less studied.

Herein, the deprotonation mechanism of phenolic groups in PG and to clarify the PG-metal complex structure were investigated. Such issues for the complexes as the chelating sites, the stoichiometric ratios, apparent formation constants (log*K*) and UV–Visible (UV–Vis) spectra were then elucidated. Besides, a fluorescence quenching method was developed to quantitatively explore the metal-binding capacities of two well-characterized polyphenolic compounds catechin (CA, Fig. 1D) and epicatechin_16_ (4 → 8) catechin (EC_16_-C), also known as PC (Fig. 1E). Consisting of 17 catechin units, EC_16_-C was often used in Hagerman’s research for qualitative and quantitative analysis of interaction between condensed tannins with protein^23^. Additionally, the quenching effects of CA and PC by metal ions in the presence of bovine serum albumin (BSA) were evaluated by fluorescence quenching method. The binding affinities and stoichiometry of polyphenolic compounds with metal ions were also proposed in the current study. The structure–activity relationships developed in this work could be used to predict the ability of other condensed tannins to bind metal ions. The current study might facilitate to understand the effect of BSA on the reaction of polyphenolic compounds with metal ions.

## Results

### pH titration study of PG

**Figure 2:**
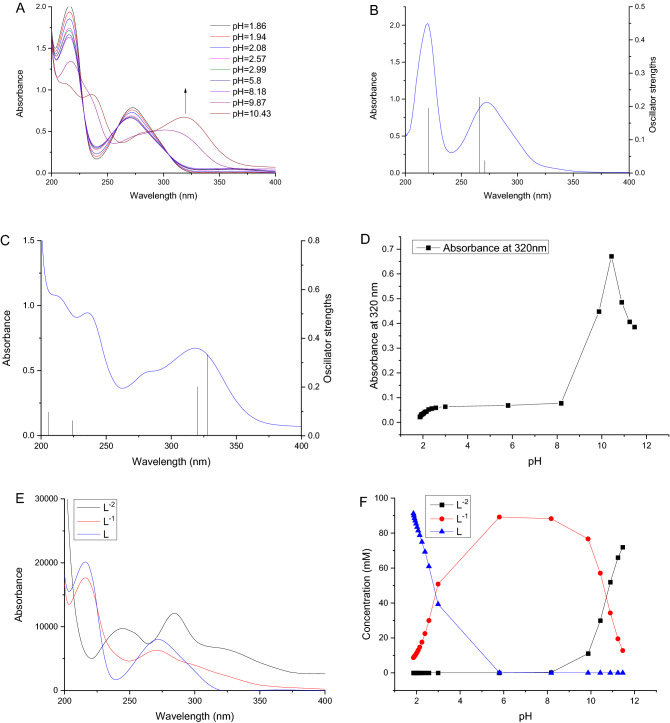
(**A**) UV–vis spectra of propyl gallate (PG) solution continuously titrated with NaOH solution; (**B**) The spectrum of the fully protonated PG was obtained at pH 1.86 (the experimental spectra are shown in blue while the computed energies of transition from the Gaussian calculations are shown as black lines); (**C**) the spectrum obtained at pH 10.43 is compared to the oscillator strength of the 9-OH quinoid PG. (**D**) Changes of absorbance at 320 nm of PG solution with increasing of pH values; (**E**) The characteristic spectra of PG (L) and its two deprotonated species (L^−1^, L^−2^) predicted using chemometric modeling method; (**F**) Concentration profiles of protonated PG and its two deprotonated species predicted by the chemometric method.

A typical UV absorption spectrum of pure PG (pH 1.86) and the variations of the spectra upon continuous titration with NaOH solution were shown in Fig. 2A. As reported in a previous study^24^, pure PG solution presented a maximum absorbance at 275 nm. Upon adding NaOH, an obvious bathometic shift of the absorbance maximum to 325 nm was observed, indicate that the phenolic hydroxyl deprotonation occurred within PG.

Bond lengths and bond angles of free and deprotonated PG were summarized in Table S1 and Table S2, respectively, and the geometrical parameters from DFT calculations were also provided. The differences of bond lengths were observed for C(5)-C(6), C(6)-C(7), C(7)-C(8), C(8)-C(9), C(9)-C(10), C(10)-C(11), and C(11)-C(6), especially for C(9)-O(15) and C(5)-O(20). By DFT calculations, the bond lengths of C(9)-O(15) and C(5)-O(20) were 1.376 and 1.209 for free molecules, and 1.237 and 1.346 for deprotonated molecules, respectively, implying the modification of structure and electron in C(9) and C(5). The bond lengths of C(8)-O(17) and C(9)-O(15) were similar in free molecules, and a decrease of 0.138 Å for the C(9)-O(15) bond length was observed. The bond angle of C(10)-C(9)-O(15) was enhanced by approximately 4.59°, and the bond angles of C(9)-C(10)-O(13) and C(9)-C(8)-O(17) in the deprotonated form were reduced by about 4.36° and 5.38°, respectively. All the above-mentioned variations were directly associated to the deprotonation mechanism of hydroxyl groups, where the rising pH removed the hydrogen bonding in free MeG molecule^13^.

The computational method was used to confirm the formation of quinoid PG in case of high concentration of NaOH. The spectra were calculated based on proposed deprotonated and quinoid PG structures by Gaussian software (Fig. 1). For different protonation states of PG and quinoid PG, the lowest energy transition wavelength and oscillator strength were computed based on the TD-DFT method of the Gaussian software. The fully protonated PG (free PG) demonstrated a maximum absorbance at a wavelength of 270 nm (Table 1), very close to the wavelength of 275 nm wavelength of the current experimental results. For the 9-OH deprotonated PG, the maximum absorbance was 310 nm (Fig. 1H), very close to the experimental result (320 nm). Figure 2B presented both experimental and computational spectra of free and deprotonated PG. The vertical line heights of computational spectra were related to the values of oscillator strengths. Due to solvent effects, the polarity of the solvent affected the electron leap, causing the actual spectrum to deviate to some extent from the theoretical spectrum. In this experiment, the difference between the calculated spectrum and the experimentally spectrum was small, which proveed the accuracy of the theoretical calculation results. The calculations showd that the band I in UV spectra of the deprotonated PG produced a significant red shift, which was consistent with the experimental results.

**Table 1:** Lowest energy transition wavelength and oscillator strength calculated for the different protonation states of propyl gallate and quinoid propyl gallate.

Considered form	Wavelength (nm)	Transition energy (eV)	Oscillator strength
PG fully protonated, A	266.97	4.6442	0.0375
PG 8-OH deprotonated, B	391.68	3.1654	0.0553
PG 9-OH deprotonated, C	310.46	3.9935	0.4596
PG 10-OH deprotonated, D	432.58	2.8661	0.0576
PG 8,9-OH deprotonated, E	663.89	1.8675	0.0000
PG 9,10-OH deprotonated, F	688.46	1.8009	0.0000
PG fully deprotonated, G	2023.20	0.6128	0.0203
PG 9-OH quinoid, H	320.35	3.8702	0.2010
PG 8,9-OH quinoid, I	629.06	1.9709	0.0000
PG 9, 10-OH quinoid, J	634.48	1.9541	0.0000

Notes: Structures are indicated in Fig. 1 F-O. All the structures were calculated using the exact same parameters with the time-dependent density functional theory level (TD-DFT) method and solvent water. PG-propyl gallate.

Previous experiments have shown that 9-OH preferred to deprotonate at higher pH in comparison with other phenolic hydroxyl in PG. In fact, when complexing with metal ions, the quinoidal form of phenolic groups was observed. In a study of anthocyanins-Al(III) complexes with a catechol moiety^25,26^, the aluminum complexes of 3′,4′7-trihydroxy-3-methoxy-flavylium chloride were found to be a quinoidal form. This form was induced by the deprotonation of two hydroxyl groups, with respect to the coordination of Al(III) to the catechol group. The hydroxyl function in position 4 of catechol group was found to be conjugated to the 4-carbonyl group, resulting in a quinoidal form by deprotonation^13^. Again, our results verified the occurrence of this phenomenon.

Figure 2C showed the consistency of the calculated data with the experimental plots. The experimental spectra are shown in blue while the oscillator strengths. from the Gaussian calculations are shown as black lines. The line heights of theoretical spectra are relative to the value of oscillator strengths. According to the computation data, the main compound category at pH 10.43 was the 9-OH quinoid PG(Fig. 2C).

Polyphenol (tannin) compounds are observed to be progressively oxidised and discoloured at different pHs, with the higher the pH of the solution the higher the degree of oxidation of the polyphenol. In a previous study, we revealed the process and mechanism of phenolic hydroxyl oxidation in polyphenols by combining computational chemistry with experimental spectroscopy. Phenolic hydroxyl groups are more prone to deprotonation in alkaline environments, which is a key process in the oxidation process. The deprotonated hydroxyl group forms an electron-rich centre, making it easier for oxygen in solution to oxidise it, resulting in the formation of quinones^27,28^. The results were also confirmed by other researchers^29^. Therefore, the main deprotonation species were the 9-OH deprotonated PG, which could be oxidized to 9-OH quinoid PG at higher NaOH concentrations.

### Calculation of pKa values of PG

Figure 2D showed the pH values and the changes of absorbance at 320 nm after each titration with NaOH. The deprotonation of PG and oxidization occurred at pH 9.87 and pH 10.43, respectively. At pH below 8.18, the predominant species were fully protonated PG, whereas deprotonated PG was not found. More PG molecules were deprotonated in case of increased pH. Nevertheless, higher pH (10.6) may form quinoid PG due to an oxidization of phenolic hydroxyl. There was a rapid increase in the absorbance of PG solution at 320 nm at pH above 8.18, which was attributed to deprotonated phenolic hydroxyl in PG. A sharp decline of absorbance was observed at pH above 10.43, indicating the hydrolysis of PG at such high pH. The p*K*_a_ of PG was calculated by chemometric modeling of the titration data. For PG, the first and second acid dissociation constants (i.e. p*K*_a1_ and p*K*_a2_) were 2.88 ± 0.05 and 10.71 ± 0.02, respectively. Moreover, the second one was very similar to that of methyl gallate (10.78 ± 0.06) reported in our previous work^27^.

### Chemometric modeling study of deprotonated PG

According to the above-mentioned spectral variations of pH titration and the theoretical data, at least two deprotonation processes of PG were proposed during the titration. Note that each p*K*_a_ corresponded to one kind of spectral species. The characteristic spectra of protonated PG species (L) and corresponding two deprotonated forms (L^−1^, L^−2^) were predicted by the chemometric modeling method (Fig. 2E). The predicted spectra were in good agreement with the experimental data. The concentration profiles of free and deprotonated PG species were also estimated by the chemometric modeling method (Fig. 2F), and only singly deprotonated species (L^−1^) were observed at pH 5.8 and the second deprotonated one (L^−2^) appeared at pH above 8.0. These results were in good accordance with the titration experiment data. It's noteworthy that approximately 12.8% of singly deprotonated species (L^−1^) of PG and around 72% of double deprotonated species (L^−2^) of PG were found at the end of titration. Therefore, the predominant species in the solution were double deprotonated species (L^−2^) of PG.

### Metal ions titration study of PG

**Figure 3:**
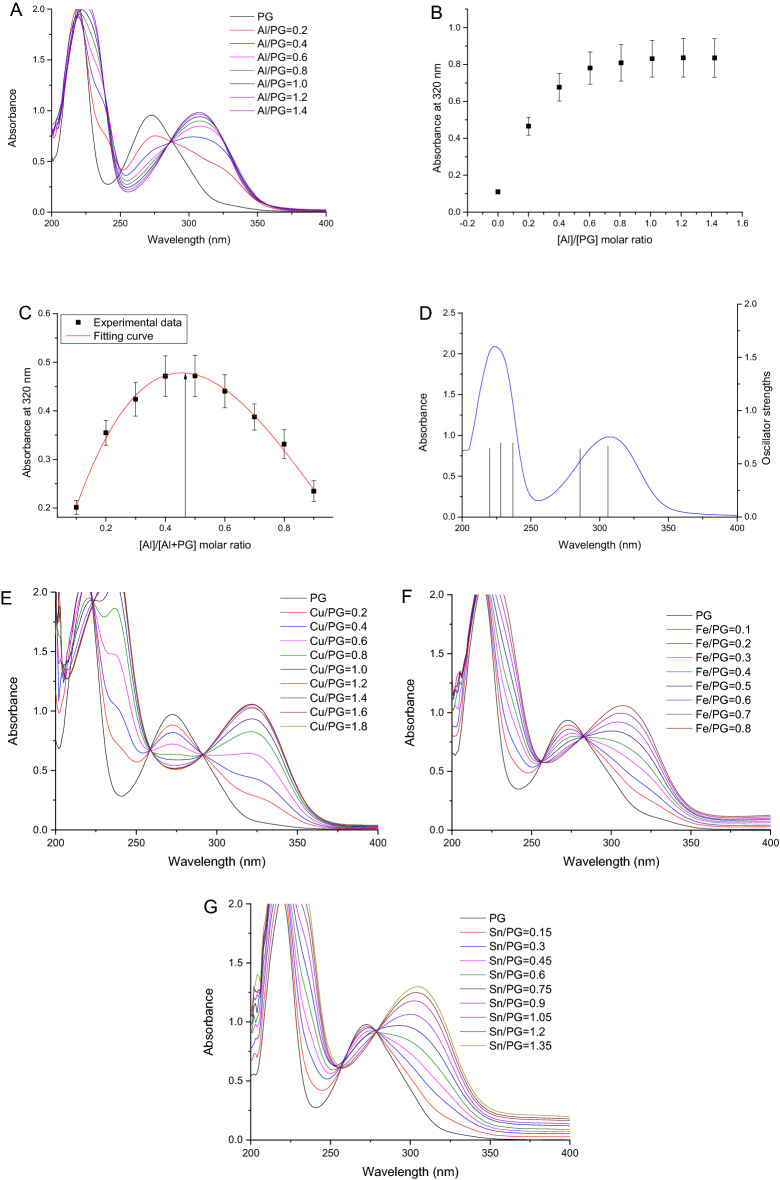
(**A**) UV–Vis spectra of 1 mL 100 µM PG titrated with 10 mM Al^3+^ solution in 2 μL increment at pH 6 acetate buffers; (**B**) Changes of absorbance at 320 nm of PG binding Al^3+^ in pH 6; (C) Experimental results of Job’s method studying Al^3+^ and PG complexation at pH 6; (**D**) Computed transitions (vertical line) and experimental spectrum (curve) for free 1:1 PG-Al complex; (**E**–**G**) UV–Vis spectra of 1 mL 100 µM PG titrated with 10 mM Cu^2+^, 5 mM Fe^3+^ or Sn^2+^ solution in 2 or 3 μL increment at pH 6 acetate buffers.

Figure 3A presented the UV spectra of PG in the buffer at pH 6.0 and PG-metal complex upon continuous titration of with AlCl_3_ solution. The spectrum of free PG in methanol was featured by a UV absorbance at 270 nm accompanied with a shoulder band at 215 nm. The addition of Al^3+^ resulted in a newly generated band of high wavelength, which was a concrete confirmation of the PG and Al^3+^ interaction. It's generally accepted that such spectral shifts were attributed to the chelation of metal with molecule bearing benzene rings^19–21^. An isobestic point was found at 286 nm, implying the existence of a single kind of complex. When the molar ratios of [Al^3+^]/[PG] was increased from 0 to around 1.0, this new band shifted toward long wavelengths with increasing characteristic absorbance at 320 nm. When the molar ratios of [Al^3+^]/[PG] was larger than 1, the absorbance at 320 nm reached a plateau (Fig. 3B), implying a maximum complexation of PG-Al complex with a stoichiometry of 1:1 at pH 6. Accordingly, the complexing of PG with Cu^2+^, Fe^3+^, or Sn^2+^ at pH 6 was also studied by UV–Vis spectra (Fig. 3E–G). The complexation between PG and metal ions was also verified by the characteristic shifts of spectral absorbance maximum to higher wavelengths.

When the ratio of [Al^3+^]/[PG + Al^3+^] was equal to 0.47 in an acetate buffer at pH 6 in Fig. 3C, the molar ratio of [Al^3+^]/[PG] was around 1:1. Under present experimental conditions, one PG molecule were solely bound to one Al^3+^ ion based on the Job’s method. Moreover, the stoichiometry of the PG-metal (Cu^2+^, Fe^3+^, or Sn^2+^) complexation at pH 6 was 0.54, 0.47, or 0.47, respectively. Therefore, all each of the four metal ions used in this study could only bind one PG molecule at pH 6.

The apparent formation constants (log*K*) for PG complexation with Al^3+^, Cu^2+^, Fe^3+^, or Sn^2+^ at pH 6 were estimated to be 5.10 ± 0.16, 5.58 ± 0.05, 4.78 ± 0.07, or 4.05 ± 0.02, respectively. The binding affinity of metal ions with PG decreased in the following order: Cu^2+^  > Al^3+^  > Fe^3+^  > Sn^2+^. The characteristic spectra of free PG species and corresponding PG-metal complex species was predicted with the chemometric modeling method (Fig. 5), and a good agreement between predicted and experimental data was obtained.

### Computational analysis of PG-Al complex

Figure 1B showed the hypothesized PG-Al complex structures for computational analysis. Figure 3D provided calculated and experimental electronic spectra of PG. The vertical lines depicted the energies from the TD-DFT method when water was used as solvent. The good matching between experimental and calculated spectra of free PG demonstrated the accuracy and validity of the method. The theoretical transition positions agreed well with the experimental spectra of complexes. Therefore, the complex structure was determined to be a formula of [Al(PG)(H_2_O)_2_Cl_2_]^-^, where Al^3+^ was coordinated at 8,9-OH doubly deprotonated catechol site with double H_2_O and double Cl^-^ ions.

By TD-DFT method, the parameters for structure (such as dihedral angles and bond lengths) were computed for PG molecule and PG-Al complex (Table S1 and Table S2). The distinct changes of benzene ring structure were revealed by comparing the structural parameters of free PG with PG-Al complex. In the complex structure, an obvious increase (from 1.379 Å to 1.481 Å) in the bond length of C(8)-C(9) was observed, due to a steric hindrance effect induced by the coordination of galloyl moiety with Al. Moreover, an obvious decrease in the bond lengths of C(9)-O(15) and C(8)-O(17) was seen, and the bond lengths of two C-O were very close (1.460, 1.453). A marked decrease in the valence angle of C(8)-C(9)-O(15) and C(9)-C(8)-O(17) was also found. Regarding the structure of ligand, the electronic delocalization was considered to lower to a small extent along the chain. However, very small variation in the bond length of C(5)-O(20) was obtained between the ligand (1.209) and corresponding Al complex (1.205), implying that the complexation rarely had some effects on the carbonyl group.

### Fluorescence of quenching spectra and quenching mechanisms of CA and PC by metal ions

**Figure 4:**
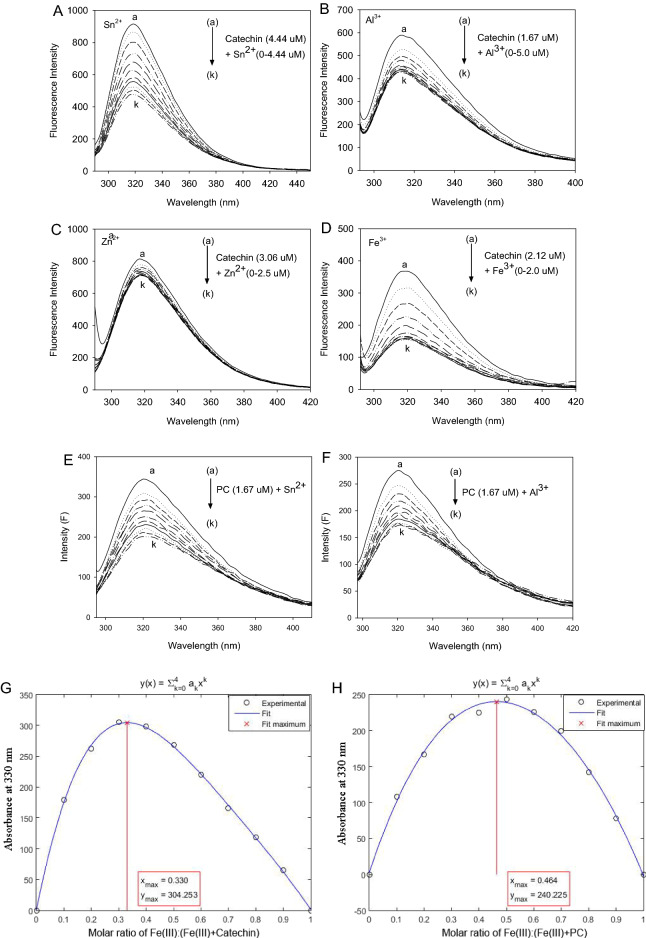
The fluorescence quenching spectrum of catechin at various concentrations of (**A**) Sn^2+^, (**B**) Al^3+^, (**C**) Zn^2+^ and (**D**) Fe^3+^, λ_ex_, 280 nm; The fluorescence quenching spectrum of PC at various concentrations of (**E**) Sn^2+^ and (**F**) Al^3+^. λ_ex_, 280 nm;* c*(PC), 1.67 μM; *c*(metal ions) (a → k), 0.0, 0.05, 0.1, 0.15, 0.2, 0.25, 0.3, 0.35, 0.4, 0.45, 0.5 μM; The binding stoichiometry of (**G**) catechin and (**H**) PC with Fe^3+^ (Using Job’s method).

The fluorescence emission spectra of CA and PC with metal cations (Sn^2+^, Zn^2+^, Cu^2+^, Al^3+^ and Fe^3+^) with varying concentrations were measured (Fig. 4). The decreased fluorescence intensities of CA and PC was accompanied by increasing concentration of metal cations. Moreover, no significant λ_ex_ shift was found after adding metal ions, indicating that the inner fluorescence of both CA and PC could be quenched by the metal cations. Therefore, the interaction between metal ions and condensed tannins was confirmed in the current system.

### Determination of binding constant (K_a_) and complexation stoichiometry of CA and PC with metal ions

The quenching constants (*K*_sv_) were calculated for the PC (or CA) and metal cations interaction (Table 2). Among all the metal quenchers, Fe^3+^ demonstrated a more powerful quenching abilities for both CA and PC, with larger quenching constants (*K*_sv_) for Fe^3+^ than those for other metals. Zn^2+^ showed the minimum quenching constant. Calculated from the modified Stern–Volmer plots, the metal binding affinities (*K*_a_) with different divalent and trivalent metal cations in the order of Sn^2+^ > Al^3+^ > Cu^2+^ > Zn^2+^ > Fe^3+^ for PC and Sn^2+^ > Al^3+^ > Zn^2+^ > Fe^3+^ for CA. The results implied the same order of metal binding affinities for PC and CA with the highest value for Sn^2+^ and the lowest for Fe^3+^. This finding was consistent with the study on the interaction between *N*,5-bis(4-chlorophenyl)-3-propan-2-yliminophenazin-2-amine (an antimycobacterial drug) and divalent metal cations by fluorescence quenching spectra^31^.

**Table 2:** Stern–Volmer quenching constants (*K*sv) and the apparent binding affinities (*K*a) for the interactions of PC and catechin (CA) with metal ions.

Polyphenols	Metal ions	pH values	*C*_(metal ions)_ (μmol L^-1^)	Complex characteristic wavelength (nm)	Complexation stoichiometry (metal:polyphenols)	*K*_sv_ (× 10^6^ L mol^-1^)	R	SD	*K*_a_ (× 10^6^ L mol^-1^)	R
PC	Cu^2+^	6	≤ 1.0	320	2:3	0.25	0.9997	0.020	2.15	0.9991
	Zn^2+^	6	≤ 1.0	320	2:3	0.21	0.9997	0.019	1.61	0.9892
	Sn^2+^	6	≤ 0.2	320	1:1	1.61	0.9998	0.084	6.77	0.9936
	Al^3+^	6	≤ 0.2	320	2:3	1.71	0.9998	0.077	5.70	0.9955
	Fe^3+^	6	≤ 0.8	320	1:1	1.42	0.9999	0.020	1.49	0.9985
CA	Sn^2+^	6	≤ 1.8	320	1:2	0.19	0.9998	0.009	1.90	0.9993
	Zn^2+^	6	≤ 1.0	320	1:1	0.12	0.9999	0.011	1.28	0.9977
	Al^3+^	6	≤ 2.0	320	2:3	0.17	0.9997	0.011	1.77	0.9955
	Fe^3+^	6	≤ 0.8	320	1:2	1.04	0.9998	0.029	0.37	0.9983

*R* is the correlation coefficient. SD is the standard deviation.

From the Job’s plot (Fig. 4) for the metal-tannin complex, it was found that the maximum changes in the relative fluorescence intensity (*F*_0_-*F*) of both complexes (CA-Sn^2+^ and CA-Fe^3+^) occurred at 0.33 molar fraction of metal ion: (metal ion plus CA) (Fig. 4G). Thus, the binding stoichiometry was 1:2^32^, that is, one molecule of metal ion bound to two molecules of CA. However, the maximum change in the relative fluorescence intensity of both complexes (PC-Sn^2+^ and PC-Fe^3+^) occurred at the ratio of metal ion to PC is 1:1 in Fig. 4H. Thus, the binding stoichiometry was 1:1, that is, one molecule of metal ion bound to only one molecule of PC. The binding affinities (*K*_a_) of Al^3+^, Sn^2+^ and Fe^3+^ (1.49 × 10^6^–6.77 × 10^6^ L·mol^−1^) with PC were much larger than those with CA (0.37 × 10^6^–1.9 × 10^6^ L·mol^−1^), indicating that the binding of metal ions with PC was much stronger than that with CA. Using the same method, both the stoichiometries of CA-Al^3+^ and PC-Al^3+^ complexes were calculated to be 2:3, that is, two molecules of Al^3+^ bound to three molecules of CA or PC. However, the binding affinity of Al^3+^ with PC (5.70 × 10^6^ L·mol^−1^) was much bigger than that with CA (1.77 × 10^6^ L·mol^−1^). In a study on the metal-phenolic complexes^33^, the difference in the coordination of ligands was supposed to originate from the availability of phenolic oxygens in phenolic compounds. It is noteworthy that the different metal-to-ligand stoichiometries were attributed to the different structures as reported by Kawabata et al.^34^.

### Effect of BSA on the polyphenols binding with metal ions

**Figure 5:**
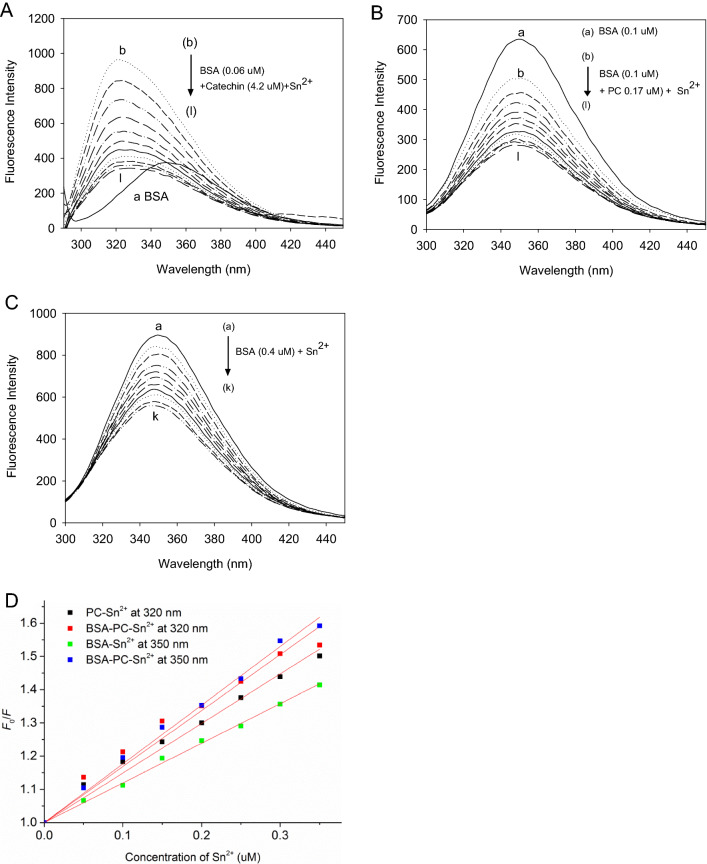
The fluorescence quenching spectrum of (**A**) catechin and (**B**) PC at various concentrations of Sn^2+^ in the presence of BSA; (**C**) The fluorescence quenching spectrum of BSA with Sn^2+^. λ_ex_, 280 nm; *c*(Sn^2+^) (**A**) (b → l), 0.0, 0.4, 0.8, 1.2, 1.6, 2.0, 2.4, 2.8, 3.2, 3.6, 4.0 μM;* c*(Sn^2+^) (**B**) (b → l), 0.0, 0.05, 0.1, 0.15, 0.2, 0.25, 0.3, 0.35, 0.4, 0.45, 0.5 μM;* c*(Sn^2+^) (**C**) (a → k), 0.0, 0.05, 0.1, 0.15, 0.2, 0.25, 0.3, 0.35, 0.4, 0.45, 0.5 μM; (**D**) the Stern–Volmer plots for PC and BSA-PC mixture fluorescence quenching by Sn^2+^ at 320 nm, and BSA and BSA-PC mixture fluorescence quenching by Sn^2+^ at 350 nm. λ_ex_, 280 nm; *c*(PC), 1.67 μM; *c*(BSA), 0.4 μM; BSA (0.1 μM) plus PC (0.17 μM).

From the fluorescence quenching spectra of CA and PC (Fig. 5 A, B) at varying concentrations of Sn^2+^ in the presence of BSA, it was found that Sn^2+^ could still bind with CA and PC in the presence of BSA. The Stern–Volmer plots of PC and BSA-PC complexes fluorescence quenching by Sn^2+^ at 320 nm and 350 nm were shown in Fig. 5D. The BSA-PC mixture had a higher binding affinity (7.50 × 10^6^ L mol^−1^) than free PC (6.77 × 10^6^ L mol^−1^) with Sn^2+^ (Table 3). However, the BSA-CA mixture had a much lower binding affinity (0.14 × 10^6^ L mol^−1^) than CA with Sn^2+^ (1.9 × 10^6^ L mol^−1^). There is no new complex formed in the reaction of CA with Sn^2+^ in the presence of BSA due to the unchanged wavelength of maximum absorption. However, the wavelength of maximum absorption of PC was changed from 320 to 350 nm in the presence of BSA, which was similar to the *λ*_max_ of the BSA-Sn^2+^ system (Fig. 5C). This suggests that the BSA-PC complexes were formed upon the addition of BSA, and the complexes could further bind metal ions, as evidenced by the fluorescence quenching spectra. PC enhanced the complexing strength of BSA with Sn^2+^, since the binding affinities increased from 2.18 × 10^6^ L·mol^−1^ for PC to 3.36 × 10^6^ L·mol^−1^ for PC. Similar results were obtained when reacting PC with Al^3+^, Fe^3+^, Zn^2+^ and Cu^2+^ (data not shown) in the presence of BSA. Therefore, it was proposed that BSA could enhance the complexing strength and chelating abilities of PC with metal ions, and that PC could also improve the complexing strength of BSA with metal ions.

**Table 3:** Stern–Volmer quenching constants (*K*_sv_) and binding affinities (*K*_a_) for the interactions of catechin (CA) and PC with Sn^2+^ in the presence of BSA.

	Wavelength (nm)	*c*(Sn^2+^) (μmol L^−1^)	*K*_sv_ (× 10^6^ L mol^−1^)	Binding affinity, *K*_a_ (× 10^6^ L mol^−1^)	*R*
[BSA-PC]-Sn^2+^	320	≤ 0.5	1.51	7.50	0.9932
	350	≤ 0.5	1.68	3.36	0.9998
[BSA-CA]-Sn^2+^	320	≤ 4.0	0.48	0.14	0.9995

*R* is the correlation coefficient. SD is the standard deviation.

The strongest binding capability was found in the cases of Al^3+^ and Fe^3+^ cations for PC, much higher than that of other metal cations. The selective coordination of Al^3+^ and Fe^3+^ with polyphenolic compounds was partly attributed to Pearson’s hard-soft-acid–base (HSAB) principle. For the HSAB principle, “hard [Lewis] acids prefer to bind to hard [Lewis] bases and that soft [Lewis] acids prefer to bind to soft [Lewis] bases to give complexes”. The cations Al^3+^ and Fe^3+^ are harder acids than Cu^2+^ and Zn^2+^ ^35^. As hard bases, the Lewis bases (oxygen-donor) located on polyphenols may be responsible for the chelation of Al^3+^ and Fe^3+^ cations. Although the phenolic group in the protonated form is not a proper ligand for binding metal cations, the deprotonated form has ideal chelating ability due to a generated oxygen center with a high charge density^14^.

The spectrophotometric titration on Fe(III)-epicatechin dimmer (B2) solutions at pH 6.9 confirmed the formula Fe(B2)_2_ for the four-coordinated species^36^. The λ_max_ and ε_max_ values of Fe(B2)_2_ were consistent with those for bis-catecholate complexes with four co-ordinations. Similar results were obtained for Fe^3+^ binding with CA. Based on the complexation stoichiometry (metal: tannin), a bis-catecholate complex of Fe^3+^-CA was proposed at pH 6 (see Graphical Abstract).

## Conclusion

The deprotonation mechanisms for the hydroxyls of the galloyl group within PG and corresponding complexation with metal ions (Al^3+^, Fe^3+^, Sn^2+^ or Cu^2+^) were studied. The predicted spectra for free and deprotonated PG models by quantum chemical calculations were in good agreement with the UV–Vis absorption spectra by experimental spectrophotometry. Higher pH (10.9) resulted in the phenolic hydroxyl oxidization, producing 9-OH quinoid PG. The first and second acid dissociation constants (p*K*_a1_ and p*K*_a2_) for PG were estimated by the chemometric modeling method. Under the conditions of pH 6.0, a stoichiometry of 1:1 was obtained for PG-metal complex. The binding affinity of metal ions with PG decreased in the following order: Cu^2+^  > Al^3+^  > Fe^3+^  > Sn^2+^. The featured spectra of free PG molecule and corresponding PG-metal complexes were predicted. Within the PG-Al complex [Al(PG)(H_2_O)_2_Cl_2_]^-^, Al^3+^ was coordinated at the 8,9-OH doubly deprotonated catechol site with double H_2_O and double Cl^-^. Moreover, a simple fluorescence quenching method was developed to study the metal chelating capabilities of condensed tannins. The complexing strength and coordinating abilities for Sn^2+^, Zn^2+^, Cu^2+^, Al^3+^ and Fe^3+^ of condensed tannins were carefully compared. The metal ions were able to quench the inner fluorescence of condensed tannins. The binding constants and binding stoichiometries of polyphenolic compounds with metal ions were determined. The binding affinities of metal ions with PC were much bigger than those with CA, implying a much stronger metal ions binding with PC than that with CA. The presence of BSA could enhance the complexing strength and chelating abilities of PC with metal ions. Based on the structure–activity relationships achieved in the present study, the properties of other condensed tannins to bind metal cations could be predicted.

## Materials and methods

### Reagents and apparatus

PG, CA, AlCl_3_·2H_2_O, CuCl_2_·2H_2_O, FeCl_3_·6H_2_O, ZnCl_2_, and SnF_2_ were obtained from Sigma-Aldrich company. Methanol, hydrochloric acid (HCl), sodium acetate (CH_3_COONa·3H_2_O) and acetic acid (CH_3_COOH) in reagent grade were obtained from Nanjing Chemical Reagent company. EC_16_-C [epicatechin_16_ (4 → 8) catechin, *M*_r_ 4930] was purified from *Sorghum bicolor* (Moench) grain and was characterized via HPLC after phloroglucinol degradation and by ^13^C NMR. The pH of prepared acetate buffer solutions (pH 6.0, 50 mM) was adjusted with a Mettler FE20K pH meter. In the study, all the other chemicals used were of analytical grade and all the solutions were prepared with doubly distilled water.

The stock 50% methanolic solution of polyphenolic compounds (1.0 mM) and the solutions of metal salts (10 mM) in HCl solution (0.1 M) were prepared freshly. The working solutions of the polyphenolic compounds (0.5 and 0.1 mM) and those of metal ions (0.5 and 10 mM) were also prepared by diluted with water. The 10 mM of sodium hydroxide (NaOH) solution was prepared in water.

The fluorescence emission spectra were recorded in the *λ*_em_ = 290–500 nm region with the maximum excitation at *λ*_ex_ = 275 nm on a LS55 luminescence spectrometer (PerkinElmer Inc., USA). For both excitation and emission, the slit widths were set at 10 nm.

### pH titration(UV–Vis spectroscopic measurements)

The pH titrations were conducted with a pipette. In a typical process, the PG solution (1 mL, 0.1 mM) in a quartz cuvette (1 mL) with a pathlength of 1 cm was titrated with a NaOH solution (200 mM) in an increment of 10 μL at 22 °C. The p*K*_a_ value of PG was determined by adjusting the pH of PG solution to around 1.8 with appropriate dilute HCl solution and then titrating with NaOH solution. The UV–Vis absorption spectra (190–600 nm) were obtained upon each addition on an Agilent 8454 UV–Vis spectrophotometer equipped with a ChemStation Software. The pH values were monitored until the readings were stable (normally 1–3 min after titration). The measurements were completed until the pH of the solution was around 12. Such an experiment was repeated for 3 times, and the potentiometric and spectrophotometric titration data were used to compute the p*K*_a_ values of PG. With the Reactlab Equilibrium software (*JPlus Consulting* http://jplusconsulting.com/products/reactlab-equilibria/), the titration results were processed by chemometric method, and the p*K*_a_ values of PG were therefore estimated.

### Metal titration experiment(UV–Vis spectroscopic measurements)

The spectrometer was blanked with an acetate buffer (900 μL, pH 6.0) in a quartz cuvette. The UV–Vis spectrum of the pure PG compound was record upon charging the PG solution (100 μL, 1 mM). The metal solution (2 μL, 10 mM) was prepared for Al^3+^, Cu^2+^, Fe^3+^, or Sn^2+^. The titration experiments started by adding the metal solution to the cuvette and the spectra were obtained after mixing for 1 min. The titration experiments continued with the metal solution (with a ramp of 2 μL) and the spectra were obtained at each titration point. To avoid the loss of the buffer capacity, the procedure ended once 20 μL of total metal solution was consumed.

### Job’s method experiment(UV–Vis spectroscopic measurements)

The Job’s method is carried out according to Zhang et al.^37^.

### Computational methods

The compound structures were constructed by the ChemDraw module and pre-optimized by the MOPAC procedure with the default settings in the Chem3D module of ChemOffice software. The generated files were transferred into mol format and then the gjf format by GaussView software. The obtained structures were optimized at the HF/6-31G^*^ level in the Gaussian 03 software to be the local minima, and were then submitted to the calculations of UV/Vis spectra, excitation energies, and oscillator strengths with the time-dependent density functional theory (TD-DFT) method coupled with RB3LYP/6-31G^**^. The TD-DFT method was also employed to calculate the bond lengths and bond angles for protonated PG, deprotonated PG and PG-Al complex.

### Metal titration experiment (Fluorescence spectroscopic measurements)

For fluorescence measurement, the metal ions solution (1 μL, 0.5 mM) was titrated into the polyphenolic compounds solution (10 μL, 0.5 mM) in the acetate buffer solution (2990 μL, pH 6), or the metal ions solution (1 μL, 0.05 mM) was titrated into the BSA buffer solution (10 μL, 1.98 mM) plus the polyphenolic compounds solution (10 μL, 0.05 mM) in the acetate buffer solution (2980 μL, pH 6). The volume of metal ions solution was varied from 0 to 20 μL.

### Determination of binding stoichiometries(Fluorescence spectroscopic measurements)

The stoichiometries of the metal ions bound to polyphenolic compounds were obtained using the continuous variations method^38^. When the final metal-polyphenol concentration was kept constant, the fluorescence change (Δ*F* = *F*_polyphenol_-*F*_polyphenol+metal_) of a series of polyphenol-metal mixtures with different molar fraction**s** were measured. Finally, a plot (known as the Job’s plot) of Δ*F* versus molar fraction of metal ions was employed to determine the binding stoichiometries based on the maximum value of the Job’s plot.

### Fluorescence spectroscopic

When a micromolecular quencher is solely bound to some equivalent sites of a fluorescent macromolecule, the relationship between the fluorescence quenching extent and the quencher concentration can be calculated by the Stern–Volmer equation:

1 $$F_{0} /F = { 1 } + k_{{\text{q}}} \tau_{0} \left[ {\text{Q}} \right] \, = { 1 } + K_{{{\text{sv}}}} \left[ {\text{Q}} \right]$$

where *F*_0_ and *F* indicate the fluorescence intensities of the fluorophore without and with a quencher, respectively, *k*_q_ is the quenching rate constant, *K*_sv_ is the Stern–Volmer quenching constant, τ_0_ (generally 5 ns) is the average lifetime of the fluorophore without a quencher^39^, and [Q] is the quencher concentration.

Negative deviation from the Stern–Volmer equation can also be seen in those systems involving multiple fluorophores (such as BSA). Instead of linear increase with [Q] in the classic model (Eq. 1), the ratios of *F*_0_/*F* decrease at higher [Q], implying the different accessibilities of fluorophores to the quencher^40^. In the modified Stern–Volmer equation (Eq. 2), *f*_a_ is the factor for fractional accessibility to calculate the updated Stern–Volmer constant *K*_a_ for those systems containing more than one fluorophores^40^.

2 $$F_{0} /\Delta F = \, \left( {{1}/\left( {f_{{\text{a}}} \times K_{{\text{a}}} } \right)} \right) \, \times \left( {{1}/\left[ {\text{Q}} \right]} \right) \, + {1}/f_{{\text{a}}}$$

## Supplementary Information

Supplementary Information.

## Data Availability

The raw datasets used during the current study are available from the corresponding author on reasonable request.
