# Supplementary material for: A spectroscopic and quantum chemical calculation method for the characterisation of metal ions complexed with propyl gallate and procyanidins
Source: Sci Rep. 2023 Feb 20;13:2977. doi: 10.1038/s41598-023-30186-x (PMC9941574; doi:10.1038/s41598-023-30186-x)
Supplement: Supplementary file 1 — Supplementary Information. [file 41598_2023_30186_MOESM1_ESM.docx]

Supplementary Materials:

**Table S1.** Bond length (Å) calculated by the DFT method for free, 9-quinoid propyl gallate (PG) and PG-Al complex.

**Table S2.**Bond angles (°) calculated by the DFT method for free, 9-quinoid propyl gallate (PG) and PG-Al complex.

**Table S1.**

| Chemical bond | Bond length / Å | Chemical bond | Bond length / Å | Chemical bond | Bond length / Å |
| --- | --- | --- | --- | --- | --- |
| 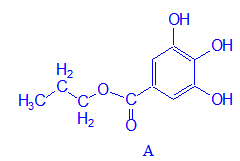 | | 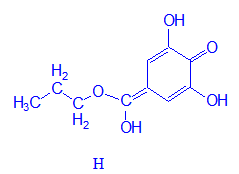 | | 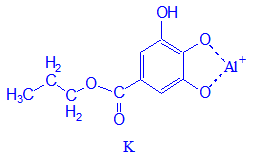 | |
| C(1)C(2) | 1.540 | C(1)C(2) | 1.540 | C(1)C(2) | 1.541 |
| C(2)C(3) | 1.523 | C(2)C(3) | 1.522 | C(2)C(3) | 1.521 |
| C(3)O(4) | 1.460 | C(3)O(4) | 1.468 | C(3)O(4) | 1.473 |
| O(4)C(5) | 1.344 | O(4)C(5) | 1.325 | O(4)C(5) | 1.331 |
| C(5)C(6) | 1.476 | C(5)C(6) | 1.352 | C(5)C(6) | 1.490 |
| C(6)C(7) | 1.387 | C(6)C(7) | 1.443 | C(6)C(7) | 1.395 |
| C(7)C(8) | 1.371 | C(7)C(8) | 1.331 | C(7)C(8) | 1.357 |
| C(8)C(9) | 1.379 | C(8)C(9) | 1.450 | C(8)C(9) | 1.481 |
| C(9)C(10) | 1.377 | C(9)C(10) | 1.444 | C(9)C(10) | 1.368 |
| C(10)C(11) | 1.380 | C(10)C(11) | 1.332 | C(10)C(11) | 1.393 |
| C(11)C(6) | 1.380 | C(11)C(6) | 1.441 | C(11)C(6) | 1.374 |
| C(11)H(12) | 1.066 | C(11)H(12) | 1.068 | C(11)H(12) | 1.067 |
| C(10)O(13) | 1.369 | C(10)O(13) | 1.375 | C(10)O(13) | 1.355 |
| O(13)H(14) | 0.968 | O(13)H(14) | 0.970 | O(13)H(14) | 0.967 |
| C(9) O(15) | 1.376 | C(9) O(15) | 1.237 | C(9) O(15) | 1.460 |
| O(15)H(16) | 0.967 |  |  | O(15)Al | 1.667 |
| C(8)O(17) | 1.383 | C(8)O(17) | 1.372 | C(8)O(17) | 1.453 |
| O(17)H(18) | 0.963 | O(17)H(18) | 0.971 | Al O(17) | 1.664 |
| C(7)H(19) | 1.070 | C(7)H(19) | 1.072 | C(7)H(19) | 1.068 |
| C(5)O(20) | 1.209 | C(5)O(20) | 1.346 | C(5)O(20) | 1.205 |

**Table S2.**

| Chemical bond | Bond angles /° | | Chemical bond | Bond angles/ ° | | Chemical bond | Bond angles/ ° | |
| --- | --- | --- | --- | --- | --- | --- | --- | --- |
| 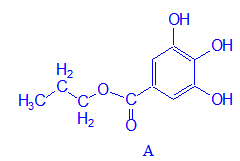 | | | 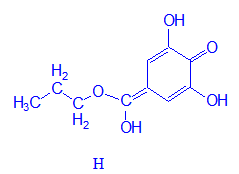 | | | 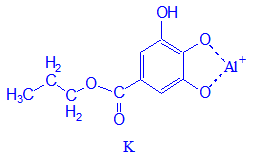 | | |
| C(1)C(2)C(3) | | 110.984 | C(1)C(2)C(3) | | 110.729 | C(1)C(2)C(3) | | 110.671 |
| C(2)C(3)O(4) | | 106.244 | C(2)C(3)O(4) | | 105.520 | C(2)C(3)O(4) | | 106.348 |
| C(3)O(4)C(5) | | 118.976 | C(3)O(4)C(5) | | 122.626 | C(3)O(4)C(5) | | 119.580 |
| O(4)C(5)C(6) | | 112.828 | O(4)C(5)C(6) | | 121.545 | O(4)C(5)C(6) | | 112.408 |
| C(5)C(6)C(7) | | 117.096 | C(5)C(6)C(7) | | 122.194 | C(5)C(6)C(7) | | 116.565 |
| C(5)C(6)C(11) | | 121.591 | C(5)C(6)C(11) | | 119.525 | C(5)C(6)C(11) | | 121.076 |
| C(6)C(7)C(8) | | 118.882 | C(6)C(7)C(8) | | 120.602 | C(6)C(7)C(8) | | 116.750 |
| C(7)C(8)C(9) | | 120.188 | C(7)C(8)C(9) | | 121.665 | C(7)C(8)C(9) | | 121.340 |
| C(8)C(9)C(10) | | 120.775 | C(8)C(9)C(10) | | 117.048 | C(8)C(9)C(10) | | 122.008 |
| C(9)C(10)C(11) | | 119.667 | C(9)C(10)C(11) | | 121.613 | C(9)C(10)C(11) | | 117.016 |
| C(10)C(11)C(6) | | 119.175 | C(10)C(11)C(6) | | 120.791 | C(10)C(11)C(6) | | 120.527 |
| C(6)C(11)H(12) | | 121.030 | C(6)C(11)H(12) | | 119.150 | C(6)C(11)H(12) | | 120.487 |
| C(10)C(11)H(12) | | 119.795 | C(10)C(11)H(12) | | 120.059 | C(10)C(11)H(12) | | 118.986 |
| C(11)C(10)O(13) | | 120.406 | C(11)C(10)O(13) | | 122.821 | C(11)C(10)O(13) | | 119.428 |
| C(9)C(10)O(13) | | 119.928 | C(9)C(10)O(13) | | 115.567 | C(9)C(10)O(13) | | 123.556 |
| C(10)O(13)H(14) | | 110.624 | C(10)O(13)H(14) | | 108.885 | C(10)O(13)H(14) | | 114.216 |
| C(10)C(9)O(15) | | 117.405 | C(10)C(9)O(15) | | 121.998 | C(10)C(9)O(15) | | 120.955 |
| C(8)C(9)O(15) | | 121.820 | C(8)C(9)O(15) | | 120.954 | C(8)C(9)O(15) | | 117.037 |
| C(9)O(15)H(16) | | 110.784 | C(9)C(8)O(17) | | 115.471 | C(9)C(8)O(17) | | 115.478 |
| C(8)O(17)H(18) | | 113.655 | C(7)C(8)O(17) | | 122.864 | C(7)C(8)O(17) | | 123.182 |
| C(9)C(8)O(17) | | 114.090 | C(8)O(17)H(18) | | 108.706 | O(15)AlO(17) | | 107.504 |
| C(7)C(8)O(17) | | 125.722 | C(8)C(7)H(19) | | 117.392 | C(8)C(7)H(19) | | 122.459 |
| C(8)C(7)H(19) | | 121.997 | C(6)C(7)H(19) | | 122.006 | C(6)C(7)H(19) | | 120.791 |
| C(6)C(7)H(19) | | 119.122 | C(6)C(5)O(20) | | 126.080 | C(6)C(5)O(20) | | 122.929 |
| C(6)C(5)O(20) | | 124.621 | O(4)C(5)O(20) | | 112.375 | O(4)C(5)O(20) | | 124.663 |
| O(4)C(5)O(20) | | 122.551 | C(5) O(19) H(20) | | 116.311 |  | |  |
| C(1)C(2) C(3) | | 110.984 | C(1)C(2) C(3) | | 110.729 | C(1)C(2)C(3) | | 110.671 |
| C(2)C(3) O(4) | | 106.244 | C(2)C(3) O(4) | | 105.520 | C(2)C(3)O(4) | | 106.348 |
| C(1)C(2)C(3) | | 110.984 | C(1)C(2)C(3) | | 110.729 | C(1)C(2)C(3) | | 110.671 |
| C(2)C(3)O(4) | | 106.244 | C(2)C(3)O(4) | | 105.520 | C(2)C(3)O(4) | | 106.348 |
| C(3)O(4)C(5) | | 118.976 | C(3)O(4)C(5) | | 122.626 | C(3)O(4)C(5) | | 119.580 |
| O(4)C(5)C(6) | | 112.828 | O(4)C(5)C(6) | | 121.545 | O(4)C(5)C(6) | | 112.408 |
| C(5)C(6)C(7) | | 117.096 | C(5)C(6)C(7) | | 122.194 | C(5)C(6)C(7) | | 116.565 |
| C(5)C(6)C(11) | | 121.591 | C(5)C(6)C(11) | | 119.525 | C(5)C(6)C(11) | | 121.076 |
| C(6)C(7)C(8) | | 118.882 | C(6)C(7)C(8) | | 120.602 | C(6)C(7)C(8) | | 116.750 |
| C(7)C(8)C(9) | | 120.188 | C(7)C(8)C(9) | | 121.665 | C(7)C(8)C(9) | | 121.340 |
